# Effects of organic materials on soil bacterial community structure in long-term continuous cropping of tomato in greenhouse

**DOI:** 10.1515/biol-2022-0048

**Published:** 2022-04-22

**Authors:** Jun Chen, Yichun Du, Wei Zhu, Xin Pang, Zhen Wang

**Affiliations:** Suzhou Polytechnic Institute of Agriculture, Suzhou 215008, People’s Republic of China; Faculty of Horticultural Science and Technology, Suzhou 215008, People’s Republic of China

**Keywords:** soil organic carbon, tomato, soil, bacterial community structure

## Abstract

Long-term fertilization will affect the above-ground vegetation, but we have little understanding of soil bacterial community structure and diversity so far. This study aims to study the effect of organic fertilization on the soil bacterial community structure and diversity of protected long-term continuous tomato cropping by using high-throughput sequencing technology. Results show that (1) fertilization application (chemical fertilizer [CF] and vermicompost [VM]) significantly changed the soil physico-chemistry properties, such as soil pH decreased compared with control treatment and increased the soil organic carbon (SOC), total nitrogen (TN), total phosphorus (TP), and total potassium (TK) contents; (2) VM increased the Shannon index of soil bacteria but decreased the soil Chao1 index; and (3) soil Proteobacteria and Actinomycetes were dominant taxa and the relative abundance of Actinobacteria increased by 36.40–44.27 and 25.80–29.35%, with CF and VM, respectively, compared with the control. Pearson correlation analysis showed that soil pH, SOC, TN, TP, and TK were the main environmental factors that affected the diversity and richness of soil bacterial communities. Redundancy analysis (RDA) showed that the SOC and TN play important roles in the composition of soil bacterial communities. In summary, the effect of VM on the soil bacterial community structure of continuous tomato cropping is better than that of CF, which should be used in the sustainable production of facility tomatoes.

## 1 Introduction

Microorganisms are a key component of the soil ecological chain. They play important roles in soil nutrient cycling, organic matter (OM) formation and decomposition, soil-borne disease occurrence and prevention, and crop growth and development. Additionally, soil microorganisms are crucial for maintaining soil ecological balance and fertility [1,2]. Therefore, maintaining high soil microbial activity and diversity is fundamental for current soil quality management practices.

Continuous protected tomato cropping is common in southern China. The continuous cropping of a single species often leads to an imbalance in the soil microbial flora, which manifests as a decrease in microbial diversity, an increase in pathogenic bacteria, and a decrease in the number of beneficial bacteria [3,4]. Furthermore, tomato production is often accompanied by excessive application of chemical fertilizers (CFs). This practice affects crop yield, soil physical and chemical characteristics, and quality and directly changes the structure of the soil microbial community [5,6]. Studies have shown that the long-term application of CFs can significantly reduce soil pH and microorganism number and diversity [6].

The use of organic materials (including livestock and poultry manure and straw) to fertilize continuous cropping soils in facilities has a high application value; it can also replace CFs and, therefore, reduce the volume used in facility agricultural production. The input of organic materials can effectively improve the structure of continuous cropping soil aggregates, increase the OM content, and reduce the salt content. Ding et al. [7] performed field experiments showing that long-term combined application of organic and inorganic fertilizers increased the diversity of soil bacteria and fungi and promoted the growth of beneficial bacteria; conversely, single application of CFs promoted fungi growth. Long-term use of organic manure and straw returning in the field can also increase the abundance of flora such as growth-promoting bacteria (plant growth-promoting rhizobacteria, which promote plant growth and many microbial products that stimulate plant growth) and arbuscular mycorrhizal fungi, which promote soil health [8,9]. However, it is still unknown whether the input of organic materials can significantly enhance the structure of the bacterial community in continuous cropping soil.

China produces billions of tons of organic solid waste every year, including agricultural waste (e.g., straw), which has surpassed 800 million tons produced yearly. A large amount of straw is burned or discarded, polluting the environment and wasting resources [10]. As of 2010, the total amount of livestock and poultry manure emissions in China has reached 1.9 billion tons, 227 million of which have not been properly treated to prevent environmental pollution [11]. This solid waste is rich in nutrients and OM needed for crop growth and development [12,13]. After being composted or adequately processed, organic solid waste can be used for fertilization in agricultural production, promoting sustainable development and reducing environmental pollution [13,14]. Therefore, in this study, we used high-throughput sequencing technology to analyze the effects of composting or earthworm treatment of livestock and poultry manure (vermicompost, VM) and CF on the composition and diversity of soil bacterial communities in facility tomato continuous cropping. We analyzed the feasibility of applying VM and CF in the sustainable production of facility agriculture and provided a theoretical basis for the rational fertilization of facility agriculture.

## 2 Materials and methods

### 2.1 Experimental design

The experiments were performed in a greenhouse in the Suzhou Polytechnic Institute of Agriculture (120.64 E, 31.43 N), Jiangsu, China, from March to June 2020. The greenhouse had natural light exposure, and the day and night temperature was controlled to be within 15–35°C.

This study used a completely random design. The fertilization treatments tested were VM, CF, and the control (CK) without fertilization. There were three treatments in total, and each was repeated five times. The total amount of N, P_2_O_5_, and K_2_O was equal in all fertilization treatments. To achieve the local general fertilization amount desired, the N, P_2_O_5_, and K_2_O dosages were set to 0.45, 0.20, and 0.40 g kg^−1^ soil, respectively (approximately 850 kg N hm^−2^, 552 kg P_2_O_5_ hm^−2^, and 850 kg K_2_O hm^−2^). The soil’s total nitrogen (TN), total phosphor (TP), and total potassium (TK) were detected by a soil elemental analyzer (FlashSmart, ThermoFisher). The soil moisture content was 43%.

The amount of VM applied was 13.10 g kg^−1^ soil (approximately 30 t hm^−2^), and the amount of RS was 45.52 g kg^−1^ soil (approximately 95 t hm^−2^) (Table 1). Additional fertilizers (urea, superphosphate, and potassium sulfate) were added to VM and RS to achieve the same N, P_2_O_5_, and K_2_O content as the CF treatment. We added the total contents of urea, superphosphate, and potassium sulfate of 50, 35, and 30 kg, respectively. The nutrient contents of the fertilizers used are shown in Table 1.

**Table 1:** The properties of VM and rice straw

Treatment	pH	SOC (g kg)	TN (g kg)	TP (g kg)	TK (g kg)	C/N
VM	6.38	2,035	46.8	17.85	4.65	7.4
RS	5.92	5,830	450.5	8.35	6.53	52.69

Note: VM: vermicompost; RS: rice straw; SOC: soil organic carbon; TN: total nitrogen; TP: total phosphorus; TK: total potassium; C/N: soil organic carbon/total nitrogen.

All fertilizers were applied as a base fertilizer simultaneously to thoroughly mix the soil and fertilizer. No top dressing was performed during the tomato growth period.

### 2.2 Soil characteristics

The soil used in this study was utilized for 5 years of continuous tomato cropping in the greenhouse. Soil pH (soil/water = 1/5) and electrical conductivity (EC, soil/water = 1/5) were measured with a pH meter (INESA, Shanghai, China) and a DDS-307 conductivity meter (Shanghai Precise Science Instrument Co., China), respectively. The OM content was estimated by the potassium dichromate dilution calorimetry method [15]. Soil’s total carbon (TC) and TN were measured using a Vario EL III element analyzer (Elementar Co., Germany) [16]. Soil TK was detected by a soil elemental analyzer (FlashSmart, ThermoFisher).

The pH value and EC of the tested soil were 6.17 and 490 μS cm^−1^, respectively, and the available nitrogen, phosphorus, and potassium contents were 206.9, 374.0, and 564.6 mg kg^−1^, respectively. The soil organic carbon (SOC) and TN content were 46.63 and 3.07 g kg^−1^, respectively, and the carbon-to-nitrogen ratio was 8.93. The tomato plants were cultivated in polyethylene pots (30 cm × 28 cm). After the air-dried soil was sieved with a 1 cm mesh, each pot was packed with 15 kg of soil with a bulk density of approximately 1.30 g cm^−3^.

The RS used in the experiment was collected from the experimental rice base of the Suzhou Polytechnic Institute of Agriculture, air-dried, and crushed to 2–3 cm before use.

On March 12, 2020, seedlings (with 3–4 true leaves) that grew sturdily, neatly, moderately, and relatively uniformly were selected and transplanted into pots. The irrigation was maintained during the growth period at 70% of the field water holding capacity and 80% of conventional field management. The test tomato variety used was “Zhongyan No. 988,” cultivated by the Beijing ZhongYanYiNong Seedling Co., Ltd. The entire growth period was 108 days.

### 2.3 Sample collection and measurement methods

Soil samples were collected during the tomato full fruit period (75 days). The soil samples were collected using a soil auger (8 cm in diameter). The soils were collected from 5 to 10 points along an S-shaped path within each treatment and mixed for a sample to ensure the representativeness of soil samples for each treatment, and immediately sent to the laboratory for analysis. A part of the fresh soil was passed through a 10-mesh sieve, and the root residues were removed and stored at −80°C for soil bacterial community and diversity analysis. Another part of fresh soil was air-dried and passed through 20- and 100-mesh sieves to determine the soil physical and chemical properties.

The total deoxyribonucleic acid (DNA) of each soil sample (0.5 g) was extracted using a Fast DNA SPIN Kit for Soil (MP Bio, USA), and the DNA was pre-checked with 1% agarose gel to assure purity. Next, the concentration and quality were tested by a NanoDrop 2000 UV spectrophotometer (ThermoFisher Scientific, USA); the qualified products were sent to Zoonbio Biotechnology Co., Ltd, to determine the composition of the soil microbial communities. Polymerase chain reaction (PCR) amplification and product purification were performed using diluted genomic DNA.

The HiFi Hotstar ReadMix PCR kit high-fidelity enzyme (KAPA Biosystems, USA) was used for PCR. PCR products were detected with 2% agarose gel electrophoresis, purified by gel extraction, and analyzed with a NanoDrop 2000 UV micro-spectrophotometer and 2% agarose gel electrophoresis to inspect the library quality. Products that passed the quality inspection were quantified with a Qubit (ThermoFisher Scientific, USA) and mixed in the corresponding proportions according to the data volume requirements of each sample.

The V3–V4 region, a highly variable region of ribosomal genes, was used for bacterial 16S rDNA amplification, and the universal primers used were F341 (5′-ACTCCTAGGGRSGCAGCAG-3′) and R806 (5′-GGACTACVVGGTATCTA-3). The index and linker sequences suitable for HiSeq2500 PE250 sequencing were added to the 5′-end of the universal primers. Amplified fragments of approximately 425 and 320 bp were obtained. After adding adapters, the products were sequenced using the HiSeq platform Illumina Miseq PE300 to obtain 2 × 300 bp Paired-End data, which was used to analyze the soil bacterial community. The Project accession number for raw data is SUB10527892.

### 2.4 Data analysis

QIIME (Version 1.7.0) was used to aggregate the 16S sequence data, and the Ribosomal Database Project (RDP) method was used for species clustering (similarity: 97%). Mothur software was used to analyze the bacterial diversity and richness index. Principal coordinate analysis (PCoA) was used in R to indicate the beta diversity of the soil bacterial community. Adonis analysis was used in R to indicate the soil bacterial community similarity. The Adonis analysis was performed by using the R software vegan package at the operational taxonomic unit (OTU) level. The OTU was used to determine significant differences between different fertilization treatments (*P* < 0.05); the data were used to construct a PCoA by using an R software vegan package at the OTU level. The Canoco 4.5 data package was used for redundancy analysis (RDA).

SPSS 17.0 (SPSS Inc., Chicago, IL, USA) was used for one-way analysis of variance and Pearson’s correlation analysis. The differences between treatments were tested with the Duncan test. The data in the graphs are all averages of five biological replicates.

## 3 Results and analysis

### 3.1 Soil physical and chemical properties

The pH of the soil treated with fertilization was significantly lower than that of CK (pH 7.20), with the most significant decrease occurring with CF (pH 4.54) and the minor decrease occurring with VM (pH 5.35) (Table 2). Compared with CK, fertilization treatments significantly increased the soil TN (6.01–26.61 g kg), SOC (10.03–15.88 g kg), TP (8.28–9.54 g kg), and TK (5.25–6.35 g kg) contents (Table 2).

**Table 2:** Soil physico-chemistry properties under different treatments

Treatment	pH	SOC (g kg)	TN (g kg)	TP (g kg)	TK (g kg)
CK	7.20 ± 0.01a	160.03 ± 6.21	20.20 ± 5.35a	8.28 ± 3.28b	5.25 ± 2.10c
CF	5.35 ± 0.02b	510.50 ± 40.85	22.35 ± 5.14a	15.35 ± 2.54a	8.78 ± 1.55a
VM	4.54 ± 0.01c	230.30 ± 5.85	22.41 ± 4.81a	9.54 ± 3.25b	6.35 ± 1.55b

CK: control; CF: chemical fertilizer; VM: vermicompost; SOC: soil organic carbon; TN: total nitrogen; TP: total phosphorus; TK: total potassium; Lowercase letter indicates the Duncan test at the *P* < 0.05 level. The table content indicates mean ± standard deviations. All values are the mean values from three replicates.

### 3.2 Relative abundance of soil bacterial groups at different taxonomic levels

**Figure 1:**
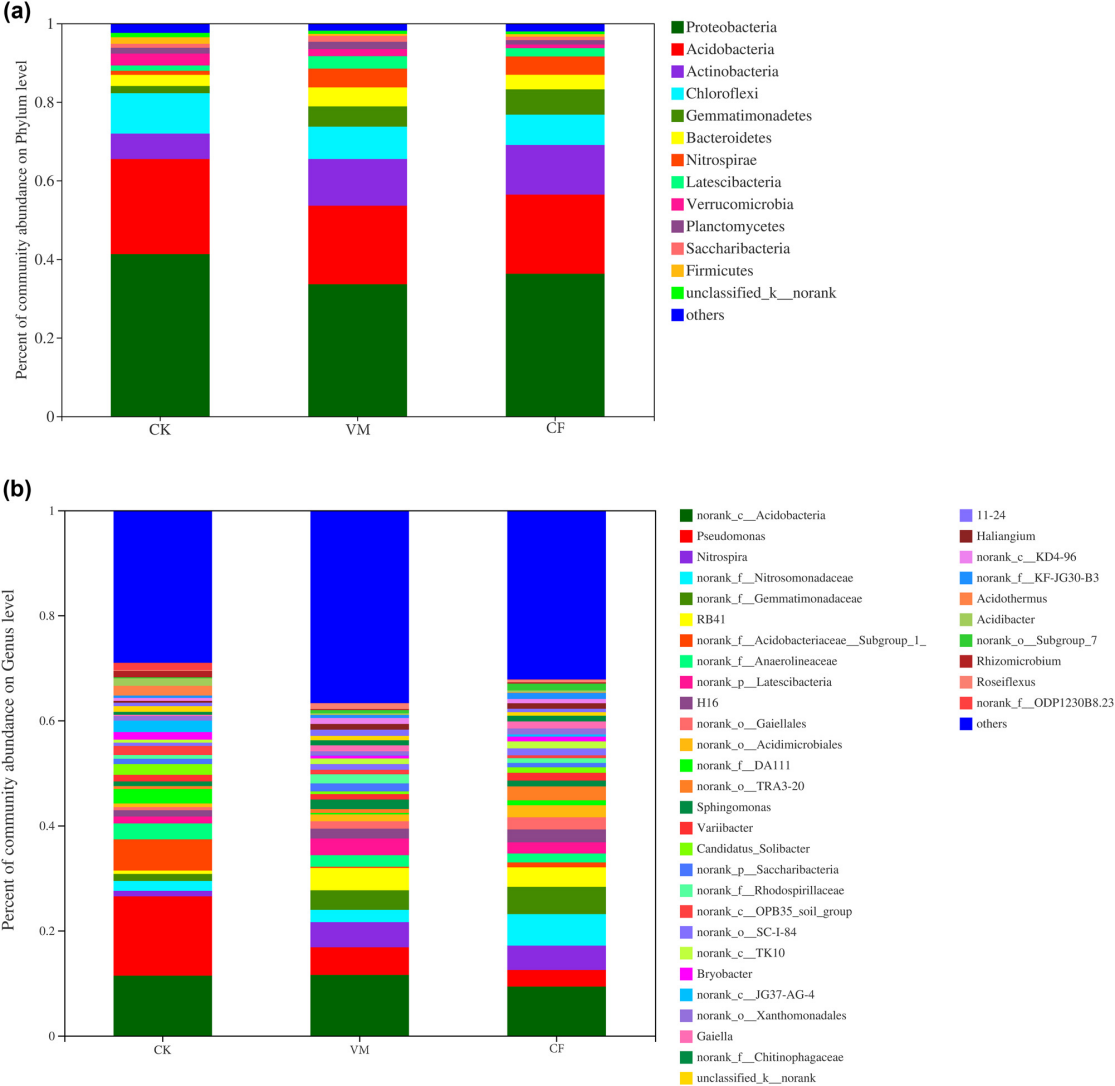
Relative abundance of soil bacterial phyla (a) and genera (b) in different treatments.

The 3153–3649 OTUs of the treated soil samples belonged to 35 phyla, 92 classes, 160 orders, 329 families, and 687 genera. At the level of bacterial phylum classification, the dominant phyla were Proteobacteria, Actinobacteria, Acidobacteria, Firmicutes, Gemmatimonadetes, Bacteroides, Candidatus Saccharibacteria, Chloroflexi, Verrucomicrobia, and Cyanobacteria (relative abundance >1%) (Figure 1). The relative abundance of the dominant bacteria groups, Proteobacteria and Actinobacteria, increased significantly after the application of organic materials (to 36.40–44.27% and 25.80–29.35%) compared with CK (35.88 and 23.10%); the relative abundance of Firmicutes and Bacteroidetes also increased significantly, but the relative abundance of Acidobacteria, Chloroflexi, Verrucomicrobiobio, Cyrobiobiosella, and Cyanobacteria was significantly reduced. CF treatments had the opposite effect on organic materials, leading to lower Proteobacteria and Actinobacteria abundances (23.46 and 19.13%). Proteobacteria had the highest abundance ratio in the CK treatment (44.27%), and the treatment with the highest abundance of Actinobacteria was VM (29.35%). These results show that the application of organic materials can increase the relative content of phylum-level dominant flora in the soil and decrease the abundance of other microbial flora to varying degrees.

Figure 1 shows the relative abundance of the bacterial genus in different treatments, in which the parts with an average abundance level lower than 0.5% are merged. They are insignificant in the CF (2.42%) treatment, compared with CK (3.65%) and VM (3.86%), slightly increasing the relative abundance of these bacteria. Gemmatimonas represented 4.72% of CM and 4.71% of VM; in CF, this dominant flora in the soil significantly reduced its relative abundance (3.29%). Gp6, Gp16, and Gp4 belong to the acid phylum (Acidobacteria), and their relative abundance is the highest in the CF treatments (8.0, 6.87, and 4.10%, respectively), followed by CK (6.33, 3.25, and 4.06%, respectively). Hence, the application of organic materials reduced the relative abundance of Gp6, Gp16, and Gp4 compared to CF and CK. Gaiella had the highest relative abundance in the VM treatment (3.78%) and the lowest in CF (2.45%). There were also several genera that changed significantly differed in abundance between different treatments. For example, Bacillus and Streptomyces had the highest abundance in CF and the lowest in VM. CK had the lowest. Porphyrobacter was significantly more abundant in VM than in CK and CF, with increases ranging 1.08–1.61 times. The relative abundance of Hyphomicrobium was the highest in VM and the lowest in CF.

**Figure A1:**
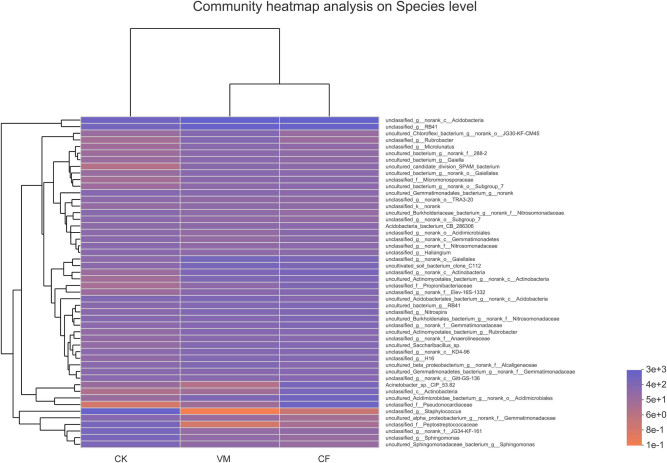
Heatmap diagram based on specie level of bacteria from the different soil samples. CK: control; CF: chemical fertilizer; VM: vermicompost.

The heatmap based on species showed the bacterial composition of different treatments (Figure A1). From Figure A3, we could infer that the bacterial compositions of VM and CF were more similar compared to that of CK, indicating that the fertilization treatment significantly changed the soil bacterial composition. Based on Figure A1, the g_staphylococcus was dominant in the VM and CF, whereas f_Peptostreptococcaceae and f_Pseudonocardiaceae were dominant in VM and CK, respectively.

### 3.3 Changes in soil bacterial community structure

The bacterial communities were analyzed by 16S rDNA sequencing of 18 soil samples obtained from 54,249 to 64,915 high-quality sequences and from 42,661 to 54,118 effective sequences (74–90% of high-quality sequences).

The average read length was 414.37 bp. The sequencing coverage rate was 97%, and a total of 3,153–3,649 OTUs were obtained, which shows that the gene sequences in the soil samples reflect the true soil bacterial community status.

**Figure A2:**
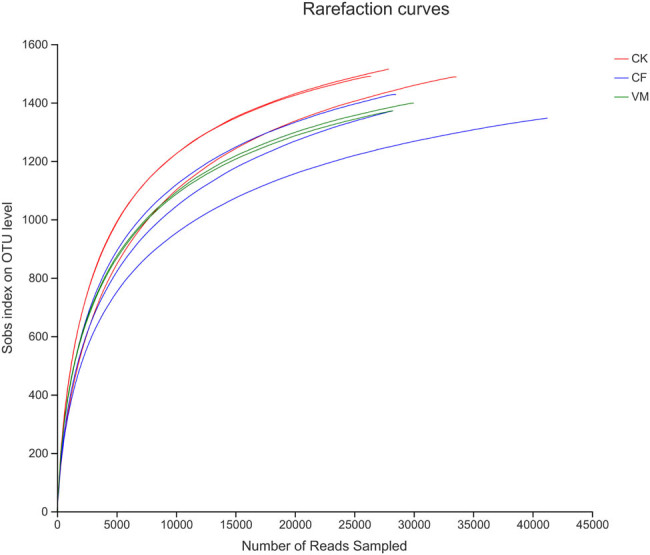
Rarefraction diagram of soil bacteria from the different soil samples. CK: control; CF: chemical fertilizer; VM: vermicompost.

The rarefaction curve indicates the sample sequencing depth and can be used to evaluate whether the sequencing volume is sufficient to cover all groups. Figure A2 shows the rarefaction curve for all samples in this test under the condition of similarity of 0.97. As shown in Figure 1, all soil sample dilution curves tended to flatten, indicating that sampling was reasonable, and the confidence in the bacterial community structure in the actual environment was high, which could reflect the bacterial community of a soil sample in a relatively realistic way (Table 3).

**Table 3:** Soil bacterial alpha diversity in different treatments

Treatments	Chao1	Shannon–weiner	Simpson	Coverage
CK	2455.45 ± 20.32b	6.54 ± 0.12b	0.005 ± 0.0001	0.99 ± 0.001a
CF	2354.20 ± 35.21b	6.32 ± 0.18b	0.008 ± 0.0002	0.98 ± 0.005a
VM	2741.35 ± 45.40a	7.86 ± 0.54a	0.009 ± 0.0001	0.99 ± 0.003a

Lowercase indicates the Duncan test at the *P* < 0.05 level. CK: control; CF: chemical fertilizer; VM: vermicompost; SOC: soil organic carbon; TN: total nitrogen; TP: total phosphorus; TK: total potassium. The table content indicates mean ± standard deviations. All values are the mean values from three replicates.

The soil Chao1 and Shannon and Simpson indices differed significantly (*P* < 0.05) between the six treatments. The Chaol index of the different fertilization modes ranked CK > VM > CF in our analyses; VM and CF treatments led to values significantly lower than CK, whereas there was no significant difference between other treatments and CK. The Shannon and Simpson indices were the highest for VM and the lowest for CF; they were significantly higher for VM than for CF treatment. The Chaol index was significantly negatively correlated with {\text{NH}}_{4}^{+}] . The Shannon index was significantly negatively correlated with EC and was significantly positively correlated with pH and TC. The Simpson index was significantly negatively correlated with EC and had a significant positive correlation with SOC (Table 4, *P* < 0.05).

**Table 4:** Pearson correlation of soil alpha bacterial diversities and soil physico-chemistry properties

	pH	SOC	TN	TP	TK	C/N
Chao1	0.385	−0.104	−0.105	−0.845	0.286	−0.82
Shannon–Weiner	0.865*	0.885*	0.203	−0.204	0.308	0.12
Simpson	0.102	0.910*	0.154	0.53	0.341	0.897*

The table content indicates the correlation coefficent by Pearson’s correlation analysis. *P < 0.05.

**Figure 2:**
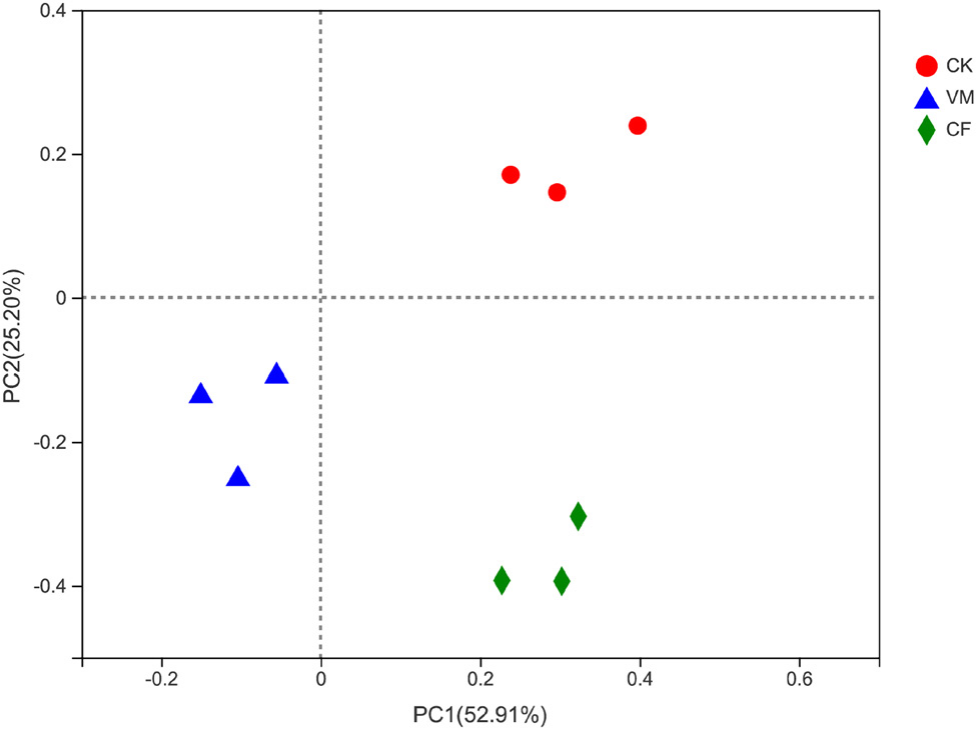
PCoA analysis of bacterial community structure in different fertilization treatments. Note: The β-diversity (changes in community structures) was calculated at the OTU level (97%) based on the Bray–Curtis dissimilarity index. CK: control; CF: chemical fertilizer; VM: vermicompost.

PCoA was used to show the soil bacterial beta diversity (Figure 2). The results showed that among all the treatments, the soil bacterial community compositions of VM and CF were the most similar.

**Figure A3:**
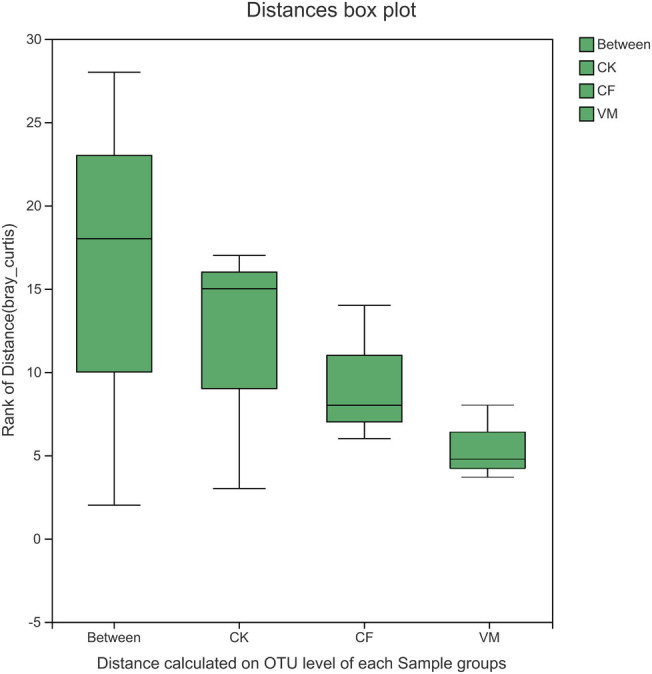
Distance box plot of soil bacteria from the different soil samples based on Adonis analysis. CK: control; CF: chemical fertilizer; VM: vermicompost.

The results showed that the first (PCoA1) and the second (PCoA2) principal component axis contributed, respectively, 18.91 and 13.81% to the difference between fertilization treatments. The distances between CF and VM were the closest, indicating that the soil OTU composition of the three was the most similar. This indicates that fertilization changed the composition of the bacterial community in continuous cropping soil, especially for the organic fertilizer treatment. The Adonis results indicate that the soil bacterial structure was significantly changed among CK, CF, and VM (Adonis *P* < 0.05, Figure A3).

**Figure A4:**
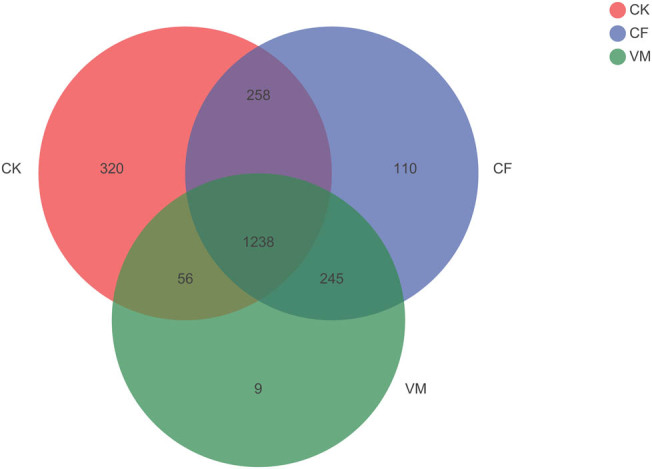
Venn diagram showing the numbers of shared and exclusive OTUs of bacteria from the different soil samples. CK: control; CF: chemical fertilizer; VM: vermicompost.

A Venn diagram illustrates the number of common and unique OTUs in three treatments, as shown in Figure A4. In total, 2,126 OTUs were detected, of which 1,238 (58.23%) were shared among the CK, CF, and VM soils (Figure A4). There were only 320 OTUs found in CK, accounting for 15.05% of the total. CF soils produced 110 OTUs, accounting for (5.17%), and the OTUs specific for VM were 9, accounting for 0.04%.

### 3.4 Correlation between soil bacterial groups and chemical properties

**Figure 3:**
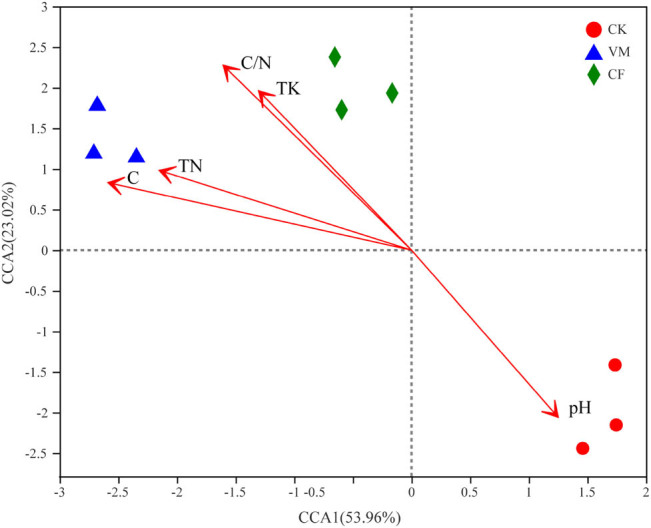
RDA of soil physico-chemistry properties and bacterial at OTU level. CK: control; CF: chemical fertilizer; VM: vermicompost.

To clarify the environmental factors that change the structure of the soil microbial community, we analyzed the correlation between the dominant flora at the bacterial phylum and the genus level and the soil chemical properties (Table 5 and Figure 3). At the phylum classification level, Actinomycetes were negatively correlated with EC and significantly positively correlated with {\text{NO}}_{3}^{-}] , {\text{NH}}_{4}^{+}] , TC, TN, and OM. Proteobacteria and Blastomonas were significantly negatively correlated with EC and significantly positively correlated with TC. The acid bacterium phylum was significantly positively correlated with EC and significantly negatively correlated with {\text{NH}}_{4}^{+}] , TC, and OM. Firmicutes were negatively correlated with pH, {\text{NO}}_{3}^{-}] , {\text{NH}}_{4}^{+}] , TC, TN, and OM; the correlation with Cyanobacteria/Chloroplast was the opposite. Furthermore, the Bacteroides phylum was negatively correlated with EC and positively correlated with {\text{NH}}_{4}^{+}] , TC, and OM; the verrucomicrobial phylum had the opposite correlations. Candidate Saccharibacteria were significantly negatively correlated with pH and positively correlated with EC, {\text{NO}}_{3}^{-}] , {\text{NH}}_{4}^{+}] , and OM. Finally, the phylum Chloroflexus was positively correlated with EC, TC, and TN (Table 5).

**Table 5:** Pearson correlation of relative abundance of soil bacterial at phyla level and soil physico-chemistry properties

	pH	SOC	TN	TP	TK	C/N
Proteobacteria	−0.214	0.803*	0.215	0.072	0.305	0.264
Acidobacteria	0.154	−0.735*	−0.284	−0.183	−0.528*	0.969*
Actinobacteiria	−0.228	0.864*	0.891*	0.314	0.872*	−0.454*
Chloroflexi	0.084	−0.682*	0.23	0.022	−0.402*	−0.204
Gemmatimonadetes	0.286	0.430*	0.136	0.801*	0.785*	0.343
Bacteroidetes	−0.028	0.785*	0.24	0.892	0.234	0.787*

*P < 0.05. The table content indicates the correlation coefficent by Pearson’s correlation analysis. *P < 0.05.

SOC: soil organic carbon; TN: total nitrogen; TP: total phosphorus; TK: total potassium; C/N: soil organic carbon/total nitrogen.

RDA results showed that the first and second ordination axes explained 56.0 and 8.6% of the bacterial community changes, respectively, and the two together explained 64.6% of the bacterial community changes (Figure 3). On the first sorting axis, the bacterial communities of organic material treatment (VM) were significantly changed compared with those of CF and CK, and they were distributed on both sides of the first axis. The overall degree of explanation of soil chemical properties for changes in bacterial community structure was 75.2%, of which {\text{NH}}_{4}^{+}] (*F* = 13.237, *P* = 0.002) and EC (*F* = 15.443, *P* = 0.002) were the most relevant, accounting for 32.1 and 24.7% of the explanatory degree, respectively. The effects of pH (*F* = 5.944, *P* = 0.004, 8.0%) and TC (*F* = 4.462, *P* = 0.008, 5.3%) were the second most important. Comprehensive analysis showed that {\text{NH}}_{4}^{+}] , EC, pH, and TC are soil physical and chemical indicators that are highly correlated with changes in soil bacterial communities.

## 4 Discussion

Soil microbial diversity is an important factor in maintaining soil health [17]. Studies have shown that continuous cropping reduces the microbial diversity around the rhizosphere and increases the abundance of pathogens, leading to the occurrence of root diseases [18]. For example, Li et al. [19] analyzed the microbial communities in rhizosphere soil samples of healthy tomatoes and diseased tomatoes suffering from bacterial wilt (*Ralstonia solanacearum*) that were grown continuously for 3 years and showed that the soil microbial diversity was higher in healthy tomato than in diseased tomato. Therefore, improving the diversity of soil microorganisms is key to alleviating soil-borne diseases in continuous cropping soil. This study showed that after fertilization with VM and CM, the soil bacterial alpha diversity increased in continuous tomato cropping and decreased after applying CFs. That might be because the application of VM improved the soil aggregate structure and chemical properties (such as pH and EC value), improving soil microbial diversity [20]. Furthermore, the increase in soil nutrients through the application of VM, especially the increase of OM (Table 2), can promote the growth of soil microorganisms [21]. Considering the chemical properties of the soil, the application of VM can significantly increase the EC value, and there is a risk of secondary soil salinization after long-term application. Hence, fertilization with earthworm manure might be a more appropriate alternative. Similar to our results, Khan et al. [22] compared the effects of long-term application of VM and inorganic fertilizer to soil used for cucumber cultivation and found that the soil microbial diversity treated was the highest with VM treatments. VM is the product of semi-decomposed agricultural or food waste that has been processed by the earthworm intestinal system. Its microbial diversity and aggregate structure are better than traditional compost [23,24]. Soils with high bacterial community diversity have functional redundancy among the communities, and the fluctuation of certain microbial groups has little effect on the overall function of soil microorganisms. Therefore, increasing bacterial community diversity can effectively maintain soil ecological functions and health [17]. The application of VM can increase the diversity of the bacterial community in the continuous cropping soil of facility-grown tomatoes, thereby improving soil disease resistance.

Ai et al. [25] found that soil disease resistance is positively correlated with the relative abundance of Proteobacteria and Actinomycota in rhizosphere soil. In rhizosphere soils with low plant incidence, the total number of these microorganisms is higher than in rhizosphere soil with high plant incidence. This may be due to the strong disease resistance of some unique microorganisms belonging to these categories [26]. We found that the relative abundances of Actinomycetes, Firmicutes, and Bacteroides were significantly increased after applying organic materials, especially VM (Figure 2), which contrasts with the results obtained by Arjun et al. [27]. This indicates that the application of VM has the potential to improve the resistance to soil pathogens. It is generally believed that the phylum Proteobacteria, which includes trophic bacteria [28], can use complex OM and plant residues as carbon and nitrogen sources [29]. Here, the content of nutrients ( {\text{NH}}_{4}^{+}] , {\text{NO}}_{3}^{-}] , TC, TN, and OM) increased after applying organic materials to the soil (Table 2), which stimulated the growth of Proteobacteria. Our results show a positive correlation between Proteobacteria and TC content, which may be because these bacteria are more sensitive to carbon nutrients [30]. Actinomycete bacteria can produce a variety of secondary metabolites (antibiotics) and extracellular enzymes that play an important role in the defense against plant diseases. Borrero et al. [31] showed that some Actinomycota bacteria have a good inhibitory effect on *Fusarium oxysporum*, the pathogen that causes Fusarium wilt. Hertweck [32] performed whole-genome sequencing on a microbial strain of the genus Ilumatobacter and found that it contained two type I polyketide synthase enzymes that synthesize polyketides, which are highly effective antibiotics. The increase in beneficial microorganisms caused by VM application may be due to the intestinal microorganisms of earthworms. Previous studies have shown that after organic materials are digested in earthworm intestines, the feces produced contain more beneficial microorganisms than the original organic materials [33]. After these beneficial microorganisms enter the soil with VM, they grow and multiply under suitable environmental conditions, promoting the defense of soil ecosystems against soil-borne diseases.

## 5 Conclusion

VM application has greater advantages than CF, RS, and conventional compost by adjusting soil pH and maintaining low soil salinity. In addition, the rich nutrient content and better soil aggregate structure in the soil VM fertilization can significantly improve the soil microbial environment and diversity, especially by increasing the abundance of some microorganisms that have an anti-pathogenic effect, which is useful to overcome the limitations of continuous cropping.

## References

[1] Zak DR, Holmes WE, White DC, Peacock AD, Tilman D. Plant diversity, soil microbial communities, and ecosystem function: are there any link. Ecology. 2003;84:2042–50.

[2] Jacobsen CS, Hjelms MH. Agricultural soils, pesticides and microbial diversity. Curr Opin Biotechnol. 2014;27:15–20. 10.1016/j.copbio.2013.09.003 24863892

[3] Li JG, Ren GD, Jia ZJ, Dong YH. Composition and activity of rhizosphere microbial communities associated with healthy and diseased greenhouse tomatoes. Plant Soil. 2014;380(1–2):337–47.

[4] Fu HD, Zhang GX, Fan Z, Sun ZP, Geng GM, Li TL, et al. Effects of continuous tomato monoculture on soil microbial properties and enzyme activities in a solar greenhouse. Sustainability. 2017;9:317.

[5] Zhong WH, Gu T, Wang W, Zhang B, Lin XG, Huang QR, et al. The effects of mineral fertilizer and organic manure on soil microbial community and diversity. Plant and Soil. 2010;326(s1–2):511–22.

[6] Hu XJ, Liu JJ, Wei D, Zhu P, Cui XA, Zhou BK, et al. Soil bacterial communities under different long-term fertilization regimes in three locations across the black soil region of northeast china. Pedosphere. 2018;28(5):751–63.

[7] Ding JL, Jiang X, Guan DW, Ma MC, Zhao BS, Zhou BK, et al. Responses of micropopulation in black soil of northeast china to long-term fertilization and crops. Sci Agric Sin. 2016;22:4408–18.

[8] Zhu F, Hou JT, Xue SG, Wu C, Wang QL, Hartley W. Vermicompost and gypsum amendments improve aggregate formation in bauxite residue. Land Degrad Dev. 2017;28(7):2109–20.

[9] Wang JY, Yan XY, Gong W. Effect of long-term fertilization on soil productivity on the north China plain. Pedosphere. 2015;25(3):450–8.

[10] Zwieten LV, Kimber S, Morris S, Chan KY, Downie A, Rust J, et al. Effects of biochar from slow pyrolysis of papermill waste on agronomic performance and soil fertility. Plant Soil. 2010;327(1/2):235–46.

[11] Fischer G, Ermolieva T, Sun LX. Environmental pressure from intensification of livestock and crop production in China: plausible trends towards 2030. Amsterdam: CATSEI; 2010. p. 1–29.

[12] Arancon NQ, Edwards CA, Bierman P, Metzger JD, Lucht C. Effects of vermicomposts produced from cattle manure, food waste and paper waste on the growth and yield of peppers in the field. Pedobiologia. 2005;49(4):297–306.

[13] Zhao J, Ni T, Li J, Lu Q, Fang ZY, Huang QW, et al. Effects of organic-inorganic compound fertilizer with reduced chemical fertilizer application on crop yields, soil biological activity and bacterial community structure in a rice-wheat cropping system. Appl Soil Ecol. 2016;99:1–12.

[14] Lazcano C, Arnold J, Zaller JG, Tato A, Domínguez J. Compost and vermicompost as nursery pot components: effects on tomato plant growth and morphology. Span J Agric Res. 2009;7:944–51.

[15] Li YJ, Wang H, Zhao JN. Effects of tillage methods on soil physicochemical properties and biological characteristics in farmland: a review. Chin J Appl Ecol. 2015;26:939–48. 26211079

[16] Xu F, Zhang T, Huai BD, Sui WZ, Yang X. Effects of land use changes on soil fungal community structure and function in the riparian wetland along the downstream of the songhua river. Environ Sci. 2021;2021(42):2531–40. 10.13227/j.hjkx.202008307 33884824

[17] Delgado-Baquerizo M, Maestre FT, Reich PB, Jeffries TC, Gaitan JJ, Encinar D, et al. Microbial diversity drives multifunctionality in terrestrial ecosystems. Nat Commun. 2016;7:10541. 10.1038/ncomms10541 PMC4738359 26817514

[18] Mareque C, Cecilia T, Beracochea M, Battistoni F. Isolation, characterization and plant growth promotion effects of putative bacterial endophytes associated with sweet sorghum (sorghum bicolor (l) moench). Ann Microbiol. 2015;65:1057–67.

[19] Li JG, Ren GD, Jia ZJ, Dong YH. Composition and activity of rhizosphere microbial communities associated with healthy and diseased greenhouse tomatoes. Plant Soil. 2014;380(1–2):337–47.

[20] Six J, Elliott ET, Paustian K. Soil macroaggregate turnover and microaggregate formation: a mechanism for c sequestration under no-tillage agriculture. Soil Biol Biochem. 2000;32(14):2099–103.

[21] Qian YL, Liang ZT, Cao Q, Yang XL, ShenYY, Wang XZ. Effects of grass-planting on soil bacterial community composition of apple orchard in longdong arid region. Chin J Ecol. 2018;37(10):3010–7.

[22] Khan MA, Shampa SA, Hossain MB. Effects of irrigation, fertilizer and manure on pore-water nutrient availability, yield and change of soil chemical properties with rice-rice cropping. Commun Soil Sci Plant Anal. 2021;4:1–12.

[23] Vivas A, Moreno B, Garcia-Rodriguez S, Benitez E. Assessing the impact of composting and vermicomposting on bacterial community size and structure, and microbial functional diversity of an olive-mill waste. Bioresour Technol. 2009;100(3):1319–26. 10.1016/j.biortech.2008.08.014 18793839

[24] Li FS, Li TL, Chang Q, Zhao FY, Yang LY. Effects of vermicomposts on tomato yield and quality and soil fertility in greenhouse under different sol water regimes. Agric Water Manag. 2015;160:98–105.

[25] Ai C, Liang GQ, Sun JW, He P, Tang SH, Yang SH, et al. The alleviation of acid soil stress in rice by inorganic or organic ameliorants is associated with changes in soil enzyme activity and microbial community composition. Biol Fertil Soils. 2015;51(4):465–77.

[26] Tamura M, Tharayil N. Plant litter chemistry and microbial priming regulate the accrual, composition and stability of soil carbon in invaded ecosystems. New Phytol. 2014;203(1):110–24. 10.1111/nph.12795 24720813

[27] Arjun S, Prasanna SD, Rameshwar T, Kanika K, Vir SR, Surender S, et al. Taxonomic and functional annotation of gut bacterial communities of eisenia foetida and perionyx excavatus. Microbiol Res. 2015;175:48–56. 10.1016/j.micres.2015.03.003 25813857

[28] Ng EL, Bandow C, Proença DN, Santos S, Guilherme R, Morais PV, et al. Does altered rainfall regime change pesticide effects in soil? a terrestrial model ecosystem study from mediterranean portugal on the effects of pyrimethanil to soil microbial communities under extremes in rainfall. Appl Soil Ecol. 2014;84:245–53.

[29] Spain AM, Krumholz LR, Elshahed MS. Abundance, composition, diversity and novelty of soil proteobacteria. ISME J. 2009;3(8):992–1000. 10.1038/ismej.2009.43 19404326

[30] Chodak M, Gołębiewski M, Morawska-Płoskonka J, Kuduk K, Niklińska M. Soil chemical properties affect the reaction of forest soil bacteria to drought and rewetting stress. Ann Microbiol. 2015;65(3):1627–37. 10.1007/s13213-014-1002-0 PMC4529456 26273241

[31] Borrero C, Ordovás J, Trillas MI, Avilés M. Tomato fusarium wilt suppressiveness. the relationship between the organic plant growth media and their microbial communities as characterised by biolog. Soil Biol Biochem. 2006;38(7):1631–7.

[32] Hertweck C. The biosynthetic logic of polyketide diversity. Angew Chem Int Ed. 2009;48:26. 10.1002/anie.200806121 19514004

[33] Gopal M, Gupta A, Sunil E, Thomas GV. Amplification of plant beneficial microbial communities during conversion of coconut leaf substrate to vermicompost by eudrilus sp. Curr Microbiol. 2009;59(1):15–20. 10.1007/s00284-009-9388-9 19280258

